# Therapeutics

**Published:** 1860-11

**Authors:** 


### THERAPEUTICS.

DISINFECTANTS.—The use of a mixture of coal-tar and plaster of Paris for purposes of disinfection and for dressing wounds, as proposed by Corne and Demeaux (*Comptes Rendus*, xlix., 127; see this Journal, xxviii., 425,) has been recently reported upon in the French Academy by a committee—Chevreul, J. Cloquet, and Velpeau (*rapporteur*)—to which the subject was referred in July, 1859.

The great interest which this method, so favorably commented upon by the distinguished surgeon Velpeau, soon after its publication, excited among the medical men of France, gave rise to the publication of numerous other systems of disinfection, which, being submitted to the Academy for its approval, were also referred to the committee in question. The labors of its members have thus been materially increased, and their report swelled to the dimensions of a general treatise upon disinfectants, especially those applicable to wounds.

In numerous experiments made at the *Hôpital de la Charité*, the mixed coal-tar and plaster of Corne was employed, both in the state of powder and as a poultice made by mixing it with oil. When applied as a thick layer, three or four times a day, upon putrid, gangrenous, and sanious wounds, the powder destroyed their odor without giving rise to any special pain. Upon indolent sores, however, or upon recent burns, the contact of the powder produced considerable smarting upon some patients, though well borne by others. Wounds of the first class were often found to be cleaned as well as disinfected; while those of the second class generally acquired a dirty, pale, gray tint, their cicatrization being hindered.

The poultices were found to be more advantageous than the powder, in the treatment of cavernous wounds, purulent or fetid, and sinuous foci, open suppurating abscesses, anthracoidal suppurations, etc.

Applied directly to the sore, the poultices destroyed the putrid odors, allayed the inflammation without augmenting the pain, leaving beneath them a healthier pus, and the surfaces in better condition. In a word, the mixed coal-tar and plaster, when properly applied, disinfects wounds and putrid suppurations. As for the absorbent and detergent qualities which its inventors also claim for it, these are less clearly evident.

The powder absorbs better than the poultices; the latter, it is true, take up a portion of the morbid exudations, but unless the dressing is carefully renewed five or six times a day, pus will nevertheless collect beneath it. From this it follows that after having been somewhat cleansed the wound ceases, at the end of a few days, to clean itself, or to heal more rapidly than it would with the usual topical applications.

Upon ulcerated cancers, the mixture, either as powder or poultice, disinfects them partially, but neither dries up the suppuration nor alleviates the pain.

It is in the dissecting-room, upon organic matter in a state of putrefaction, that the mixed coal-tar and plaster is all-powerful. The most infectious masses, when imbued with the powder, or simply rolled about in it, lose at once their disagreeable odor. According to Velpeau, his autopsy room was as approachable toward the close of last summer as it had formerly been repulsive. It was freed from flies and other insects, as well as from putrid odors.

Although it would have been out of the province of the committee to experiment upon the application of this mixture in disinfecting filth upon a great scale, they have nevertheless proved that it can be advantageously used in hospitals, for deodorizing urine or fecal matters.

The following inconveniences, to which the use of the mixture in surgery would give rise, are enumerated:—

It not only soils the clothes of the patient, but hardens them and causes them to weigh more heavily upon or about the wound; it imparts to the bandages, with which the poultices are covered, a very tenacious, rusty or yellow color; it must be frequently renewed, and although it destroys putrid smells, it retains a bituminous odor by no means agreeable to many persons.

These inconveniences are of comparatively slight importance, it is true, and may possibly admit of being remedied.

Of the other disinfectants submitted to the committee, several were only modifications of that of Corne and Demeaux. Vegetable tar, as shown by Renault, may be substituted for coal-tar. A mixture composed of hydraulic lime and tar did not disinfect wounds to which it was applied, nor could it be supported by the patients. With regard to the assertions of some practitioners, that common earth, talc, flour, or other vegetable and mineral powders, even poudrette, when mixed with coal-tar, furnish a more convenient and less costly disinfectant than that prepared with plaster, the experiments of the committee have proved that while coal-tar, mixed with common earth, well dried, or with sand, is equally, or perhaps much more efficacious for disinfecting fecal matter as when mixed with plaster; that while comparative experiments made from this point of view upon sulphate of lime, clay, charcoal, linseed meal, and earth have resulted in favor of the latter, the same is by no means true in surgery. When applied to wounds or infectious suppurations, these different mixtures were only partially successful, having proved to be less efficacious than the mixed plaster and coal-tar. In like manner the proposal to use an emulsion of coal-tar and tincture of saponine has not been found advantageous in practice; most patients complained of it, their wounds exhibited scarcely anything satisfactory, while the disinfection was very imperfect. The mixture of plaster and coal-tar was substituted for it, upon the same wounds, with decided advantage.

Although the modifications of Corne and Demeaux's process have not been particularly felicitous thus far, they have nevertheless served to confirm the fact that in reality it is the coal-tar which acts the principal part as disinfectant in these various mixtures.

Among the numerous other substances proposed as disinfectants, or for dressing wounds, the following have not afforded satisfactory results:—

*Chlorate of potash*—mixed with clay or kaolin (for example, ten parts of chlorate to ninety parts of white clay or fine sand)—which was proposed as an absolute disinfectant, neither disinfected nor absorbed the pus of fetid wounds. The mixture would be in any case much more costly than coal-tar and plaster, and certainly less efficacious.

*Whites of eggs*—mixed with chalk and applied to wounds, previously oiled, succeed no better than simple cerate.

*Powdered sugar*—when employed in layers upon ulcers, forms crusts, beneath which the suppurations accumulate and hinder the process of healing.

*Cherry-laurel water, glycerin, and cellulose.*—According to Antier, glycerin mixed with equal parts of cherry-laurel water forms a valuable absorbent or disinfectant to be applied as a lotion or injection. This mixture, converted into pomade by mixing it with powdered almonds, was also proposed as a topical application for all kinds of wounds. But in the hands of the committee neither the liquor nor the pomade, by themselves or mixed with kaolin, produced any effect more marked than that of lead-cerate and other anti-putrid or detergent solutions already in use.

The members of another group of disinfectants are worthy, in various degrees, of consideration.

Among these charcoal appears in the front rank. Surgeons have long regarded it as one of the best antiseptics known. Confined between pieces of linen, according to the process of Malapert and Pichot, it is more readily applied than when used as a powder directly upon wounds; but the mixture of coal-tar and plaster, which disinfects still better and is more cleanly, is susceptible of a simpler and a more general application.

*Coke of Boghead coal*—in powder, as proposed by Moride, like carbon when employed comparatively with coal-tar and plaster, alternately upon the same patients, proved to be less efficacious, less convenient, and more disagreeable than the latter.

*Mixed plaster and charcoal*—proposed by Herpin, of Metz, irritates the wounds, disinfects badly, and soils everything it touches.

*Carbonic acid*—proposed by the same author, appears to the committee to be too difficult of application in practice, though theoretically founded upon important analogies.

*Bituminous water of Visos*—proposed by Manne, and the mud of rivers used as a poultice by Desmartis, do not appear to be susceptible of being substituted for the mixture of Corne and Demeaux.

The following substances have long ago acquired a place, each in its own way, in the class of disinfectants.

*Tincture of iodine* has been employed as an antiseptic by hospital surgeons since 1823. By modifying the surfaces to which it is applied, it usually improves the appearance of the pus, lessens its acridity, and is, to a certain extent, antagonistic to putrid infections. It disinfects, however, only incompletely, causes severe pain when applied to open wounds, and would be expensive if used on a large scale; finally, the odor of iodine is neither agreeable nor unattended by inconveniences.

*Perchloride of iron* has been used for some twelve years in hospitals as an antiseptic and as a means of modifying certain wounds and putrid or sanguineous foci. Without diffusing the disagreeable odor of tincture of iodine, it has, like the latter, the fault of disinfecting badly, of causing much pain, and of acting violently upon the diseased tissues, besides injuring the cloths which are soaked in it even more than is the case with the coal-tar and plaster.

Both iodine and the salt of iron just mentioned are in fact agents of another order; they have rendered, and do still render important services. They are certainly well worth preserving, but should not be compared with the mixture of coal-tar and plaster.

*Nitrate of lead*, *creosote*, and other substances which have been proposed at one time or another, have not realized the expectations of their inventors; their price has been too great, their employment required too much care, or their action has not been sufficiently certain that they could be advantageously used in practice. There is, nevertheless, one of these which deserves special mention, viz., *chlorine*. Ever since Guyton Morveau demonstrated the true action of muriatic acid upon putrefying animal matters, the efficacy of chlorine has been tested in almost innumerable ways. Solutions of chlorine, of "chloride of soda," and of "chloride of lime," have rendered signal services to medicine and in the cause of public health, especially since Labarraque, some thirty years since, indicated an improved method of employing them. But the odor of chlorine, disagreeable in itself, is neither easily borne nor devoid of inconveniences. Wounds, moreover, hardly accommodate themselves to it any better than the sense of smell, whenever somewhat large doses of it are required.

*Chlorinated sponge.*—The idea of applying sponges saturated with chlorinated solutions, directly upon purulent or gangrenous wounds, as suggested by Hervieux, appears to be excellent for certain cases. Such sponges, renewed several times per day, absorb the pus gradually as it forms better than anything else, and disinfect the wound very well. Unfortunately, chlorine rapidly alters or destroys the sponges and soon causes undue irritation. While this method, therefore, is an excellent one for cleaning certain gangrenous and sinuous wounds, it is, nevertheless, inferior in most instances to the mixture of coal-tar and plaster.

*Subnitrate of bismuth*—suggested by Frémy as an absorbent and disinfectant, was applied to a large number of wounds. Upon large cavernous cancers it disinfected somewhat better than Peruvian bark, charcoal, or chlorate of potash, but less than the coal-tar and plaster. By its use, however, several bad-looking wounds were cleansed quite rapidly. Since it causes no pain or irritation, and since it neither soils the skin nor the clothes, the subnitrate of bismuth is preferable to a multitude of other antiseptic powders; but it is useful rather as a solidifier (*incarnatif*) or dryer, than as an absorbent or disinfectant.

In their *résumé* the committee affirm:—

I. That coal-tar mixed with plaster, according to the formula of Corne, (see this Journal, xxviii., 426,) can disinfect putrefying organic matters. Mixed with alvine dejections this powder destroys their odor and leads one to hope that by its aid profound reforms in the present system of maintaining and clearing out cess-pools, etc., may some day be brought about. For this purpose, ordinary earth, coal-ashes, or sand may be substituted for the plaster, as Cabanes prefers, being at least equally efficacious.

II. In therapeutics the coal-tar and plaster has fulfilled only a part of its promises. As a disinfectant in the dissecting-room, upon the folds of bandages —everywhere where there is infectious matter, its qualities are incontestable. This is also true as regards putrid or gangrenous foci, fetid suppurations, sanious wounds, ichorous putrilagenous cavities, hospital gangrene, etc.; but upon acute and exposed wounds, or upon ordinary wounds or ulcers, other topical applications are preferable to it.

III. Used with lint upon cloths, with pomades or cerate, it has afforded no useful result, and nothing has occurred to prove that when administered internally it has produced the least benefit.

IV. As an absorbent it leaves much to be desired, although it is not entirely devoid of action. When applied as a poultice, in particular, it absorbs very incompletely. For that matter the mixtures of coal-tar with earth or with other powders absorb still less than the mixture of Corne and Demeaux, and are scarcely at all applicable in therapeutics. In this connection it must not be forgotten that the morbid liquids, and particularly pus, are very different from water. A substance like plaster, for example, which absorbs water strongly, might be incapable of absorbing pus. It is nevertheless true that, as an absorbent, the mixture of coal-tar and plaster, either as powder or as poultice, is of some use upon fetid and putrid wounds or suppurations.

V. Cellulose, glycerin, and cherry-laurel water; chlorate of potash mixed with talc, clay, marl, or kaolin, are neither sufficiently efficacious, nor in application are they convenient enough to be retained in practice.

VI. The mixture of saponine and coal-tar does not appear to be preferable for dressing wounds to many other liquids already known—tincture of aloes for example. The same may be said of the mixed coal-tar and charcoal of Herpin; nor does it seem as if carbonic acid should be used, unless some improved method of applying it can be devised.

VII. The Boghead residue would be useful only in lack of coal-tar and plaster; while charcoal in porous envelopes does not mould itself to cavernous and sinuous wounds with sufficient readiness to come into general practice.

VIII. From its low price, and by its action, at once mild, absorbent, and disinfectant, as well as by its drying properties, the subnitrate of bismuth will render important services in default of the mixture of coal-tar and plaster. It is even preferable to this when the wounds are accompanied or surrounded with heat or irritation.

IX. Tincture of iodine and perchloride of iron act rather by modifying the surfaces of wounds and of purulent foci, than as absorbents or disinfectants. They have their special applications in surgery, but agents of this sort are not comparable with the mixed coal-tar and plaster.

X. Sponges soaked in chlorinated water can also render good service upon pale, burrowing sores and upon gangrenous foci.

We have occupied ourselves, say the committee, only with the practical or experimental side of the question. A discussion of its theoretical or chemical bearings would have carried us too far. Moreover, since the authors of the different communications which have been submitted to us have themselves neglected this for the most part, it has seemed to us useless to treat of it at present; whether it be the phenic acid or rosolic, or brunolic acid, or the anilin, picolin, etc., of the coal-tar, which disinfects, is in reality of but little importance. Science will inform us of this some day no doubt; for the moment we have merely to ascertain whether or no the various disinfectants which are brought to us do really disinfect.

After citing the labors of various persons who have proposed methods of disinfection, the committee go on to say: "M. Corne, and the authors indicated above, occupied themselves only with the disinfection and the solidification of animal matter, having in view the preparation of manures. * * * * It is M. Demeaux who appears first to have had the thought of applying to fetid wounds, in surgical practice, the powder invented, or adopted and extolled, by his neighbor. In addition, it is evident that here, as is the case with so many other complex facts with which science is enriched, there is, so to say, neither invention nor priority for any one. The subject has been worked upon for more than a century—a multitude of savans having competed with each other in studying it. Little by little the evolution of the discovery has been effected. M. Corne disengaged it from its gangue a little better than his predecessors, and Demeaux, knowing, perhaps, that from time immemorial sailors and the inhabitants of certain southern countries often dress their wounds with tar; that tarwater and pomades of tar are frequently employed in medicine, has extended its applications to therapeutics."

"Many other efforts are still necessary. In point of fact, the results thus far obtained are merely rough outlines—only first trials. So long as the world at large is not in possession of a simple, easy, and economical method, accessible to every one, which shall be capable of disinfecting immediately, and without inconvenience on the large or small scale, dejections and filth of all kinds, in dwellings as well as in privies or slaughter-houses, in dissecting-rooms and the like, as well as in the sick-room, upon wounds, improvements will still be wanted; there will yet be room for new attempts. While recording those of to-day and those of yesterday upon the road already traversed, let us be careful not to diminish the ardor of the laborers in the future, who will finally endow civilization with a complete and general disinfectant."

Finally, certain indispensable precautions must be followed, in order to obtain from the process of Corne and Demeaux its proper effect. It is evident, from having neglected some of these precautions, that different experimenters have been led to believe that the method is useless. Fine moulding plaster, and not the common article, should be employed. The coal-tar, which is mixed with it in the proportion of two to four parts to a hundred, by triturating or grinding, ought to impart to it a gray tint, without destroying its dry, pulverulent condition. Objects to be disinfected should be rolled in this powder until each point upon their surfaces has been brought in contact with it. Gangrenous or putrid sores should be covered with thick layers of it, by handfuls, several times per day. If one is treating pus, blood, dejections, or the like, enough of the powder should be added to form a paste of the mass, taking care to replace the first layer of powder by another as soon as it no longer absorbs any more. Mixed with oil to the consistence of a thick pap, it forms poultices of convenient application, if they are made thick and broad.

Within the limits which have been indicated the mixture of coal-tar and plaster is a good disinfectant, and may be recommended for use in domestic economy as well as in hospitals. "What we have ourselves seen leaves no uncertainty of the reality of this property, nor of the possibility of its application." * * * * * It remains only to draw from it reasonable, practical consequences, either taking the fact as it is, or by modifying and perfecting it.—(*Comptes Rendus*, l., 279; *Amer. Jour. of Science and Arts*, July, 1860.)

